# sFRP4-dependent Wnt signal modulation is critical for bone remodeling during postnatal development and age-related bone loss

**DOI:** 10.1038/srep25198

**Published:** 2016-04-27

**Authors:** Ryuma Haraguchi, Riko Kitazawa, Kiyoshi Mori, Ryosuke Tachibana, Hiroshi Kiyonari, Yuuki Imai, Takaya Abe, Sohei Kitazawa

**Affiliations:** 1 Department of Molecular Pathology, Ehime University Graduate School of Medicine, Shitsukawa, Toon City, Ehime 791-0295, Japan; 2 Department of Diagnostic Pathology, Ehime University Hospital, Shitsukawa, Toon City, Ehime 791-0295, Japan; 3 Department of Pathology, National Hospital Organization, Osaka National Hospital, Hoenzaka, Chuo-ku, Osaka City, Osaka 540-0006, Japan; 4 Animal Resource Development Unit, RIKEN Center for Life Science Technologies, Minatojima Minami-machi, Chuo-Ku, Kobe City, Hyogo 650-0047, Japan; 5 Genetic Engineering Team, RIKEN Center for Life Science Technologies, Minatojima Minami-machi, Chuo-Ku, Kobe City, Hyogo 650-0047, Japan; 6 Division of Integrative Pathophysiology, Proteo-Science Center, Ehime University Graduate School of Medicine, Shitsukawa, Toon City, Ehime 791-0295, Japan

## Abstract

sFRP4 is an extracellular Wnt antagonist that fine-tunes its signal activity by direct binding to Wnts. Bone fragility under oxidative stress by diabetes and aging is partly related to the suppression of the Wnt signal through upregulated sFRP4. Here, to explore the functions of sFRP4 as a balancer molecule in bone development and remodeling, we analyzed the sFRP4 knock-in mouse strain. X-gal and immunohistochemically stained signals in sFRP4-LacZ heterozygous mice were detectable in restricted areas, mostly in osteoblasts and osteoclasts, of the femoral diaphysis after neonatal and postnatal stages. Histological and μCT analyses showed increased trabecular bone mass with alteration of the Wnt signal and osteogenic activity in sFRP4 mutants; this augmented the effect of the buildup of trabecular bone during the ageing period. Our results indicate that sFRP4 plays a critical role in bone development and remodeling by regulating osteoblasts and osteoclasts, and that its functional loss prevents age-related bone loss in the trabecular bone area. These findings imply that sFRP4 functions as a key potential endogenous balancer of the Wnt signaling pathway by efficiently having direct influence on both bone formation and bone absorption during skeletal bone development and maintenance through remodeling.

Bone formation and resorption are strictly regulated by two types of cells, osteoblasts and osteoclasts^1^,^2^. Osteoblasts are specialized bone-matrix producing cells originating from undifferentiated mesenchymal cells^3^. By regulating proliferation, differentiation and maintenance of osteoblasts, various bone-seeking inter- and intracellular signals and hormones, cytokines, enzymes, minerals, and transcription factors are known to modulate bone mass^4^,^5^. Among these signals, bone morphogenic protein (BMP)-Smad, hedgehog and Wnt/β-catenin associated pathways (both canonical and non-canonical) are three established pathways that induce osteoblast-specific gene expression^4^,^5^,^6^. Of these, the canonical Wnt/β-catenin pathway plays a central role in bone mass by modulating both osteoblasts and osteoclasts. Indeed, loss-of-function mutation in low-density lipoprotein receptor-related protein (Lrp) 5, a co-receptor for transduction of canonical Wnt signaling, leads to a low bone mass phenotype in both humans and mice, whereas gain-of-function mutation in Lrp5 results in high bone mass in humans^7^,^8^,^9^,^10^,^11^. The Wnt/β-catenin pathway, on the other hand, is controlled by rather complicated extra-, inter- and intracellular agonists and antagonists^12^,^13^.

The secreted Frizzled-related proteins (sFRPs) comprise a family of five proteins in mammals that were first identified as antagonists of the Wnt/β-catenin pathway during embryogenesis^13^. sFRPs serve as soluble decoy receptors for the Wnt ligand, and thus antagonize both canonical and non-canonical Wnt/β-catenin pathways. One of the physiologic roles of sFRPs is thought to form a local morphogenic gradient during embryogenesis by locally antagonizing the Wnt/β-catenin pathway^13^,^14^. On the other hand, high serum levels of sFRPs have been documented in pathological conditions such as obesity, diabetes and osteoporosis^15^,^16^,^17^. In contrast, during the steps to carcinogenesis, epigenetic inactivation of sFRP genes by hypermethylation around transcription start sites, allows the silencing of all sFRP genes in human mesothelioma and colorectal cancers^18^,^19^. Among sFRPs, sFRP4 is unique in that oxidative stress derepresses the epigenetically silenced sFRP4 gene by sequestering the MeCP2 protein that is bound at the typical MeCP2-binding site (cgcgtctggataaata) located adjacent to TATA-box while keeping methylated cytosine unchanged^20^. Their *in vitro* study led to our speculation that, as a target gene for oxidative stress or aging, consequent overexpression of sFRP4 may play a noxious role in the pathogenesis of obesity, diabetic complication or aging^20^,^21^. To probe this hypothesis, we generated sFRP4 knock-in mice that provide the additional advantage of highly sensitive and easy *in situ* detection of sFRP4 gene expression as well as of the genetic gene inactivation, and observed the biological role of sFRP4 at individual level.

## Results

### Generation of sFRP4 knock-in mice

We generated an sFRP4 ^LacZ^ allele by knocking β-galactosidase (LacZ) into the endogenous sFRP4 gene (Supplementary Fig. 1a). sFRP4 ^LacZ/+^ mice were derived from mouse embryonic stem cells that had undergone homologous recombination at the sFRP4 gene locus (replacing its first exon including the start codon (ATG) with a LacZ-neo selection cassette). The targeted mutation of sFRP4 ^LacZ/+^ mice was confirmed by Southern blot and genomic PCR (Supplementary Fig. 1b,c).

### Regulation of sFRP4 expression during bone formation and remodeling

**Figure 1:**
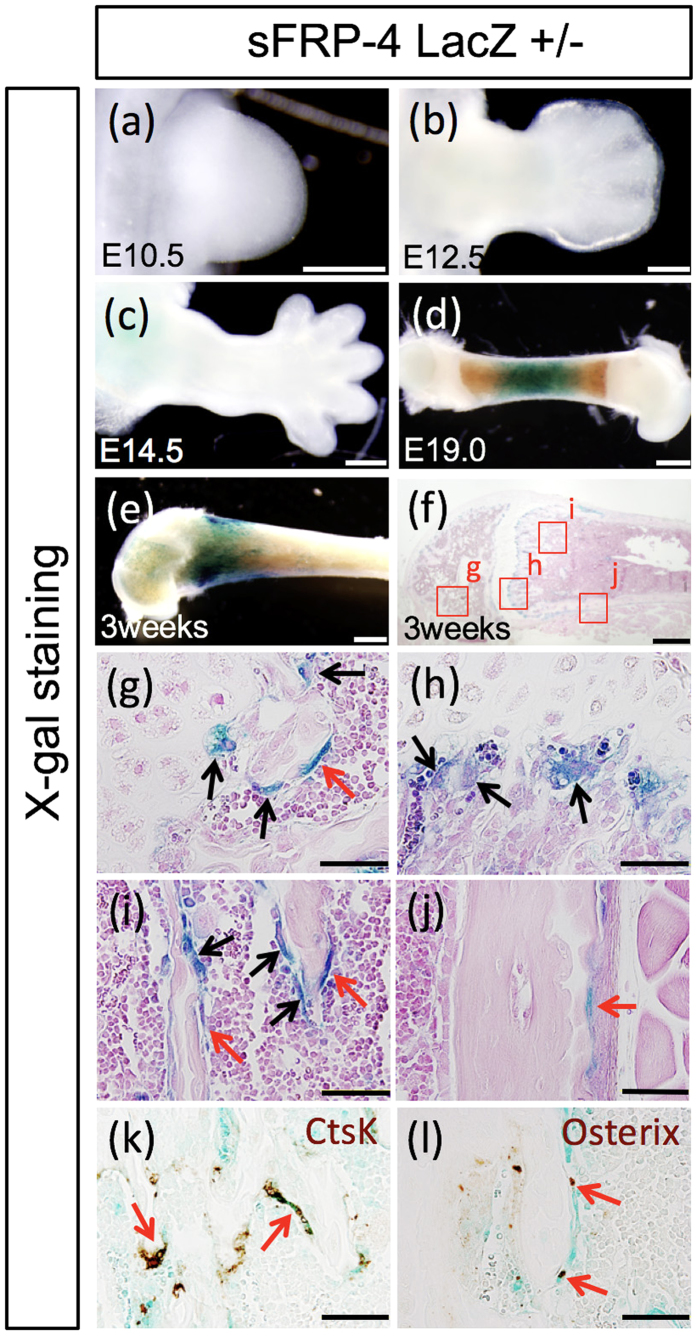
Temporally restricted manner of sFRP4 gene expression in the developing skeleton of mouse limb. Whole-mount (**a–e**) and section views (**f–l**) of X-gal staining signals in sFRP4-LacZ knock-in reporter heterozygous mice (Supplementary Fig. 1. shows the sFRP4 gene targeting construct). (**a–c**) β-galactosidase activity is not detectable in the limb bud at early- and mid-embryonic stages. (**d**) Gradual β-galactosidase activity is detected around the neonatal stage. (**g–j**) Prominent X-gal stained signals in the mouse femur at 3 weeks of age. Strong β-galactosidase activity is detected at the mononucleic flat cells and the multinucleic cells in the epiphyseal nucleus (**g**), metaphysis (**h**), diaphysis (**i**) and periosteum (**j**). The mono-nucleic flat cells (red arrows) and multinucleic cells (black arrows) are seen in (**g–j**). (**k**,**l**) Immunohistostaining of X-gal stained sections of the diaphysial region of the femur with anti-Cathepsin Ab and anti-Osterix Ab confirms that β-galactosidase positive cells are localized in the osteoclastic and osteoblastic cell lineages (red arrows). Control experiments for X-gal staining are shown in Supplementary Fig. 2. Bars 500 μm (**a–f**), 50 μm (**g–l**).

The expression of sFRP4 in osteoblastic cell lineages has been reported^21^,^22^,^23^. For further detailed histological characterization of sFRP4 gene expression during bone development, the expression pattern of the LacZ reporter gene was analyzed in sFRP4 ^LacZ/+^ mice during embryonic and postnatal development (Fig. 1). At early- and mid-gestation stages, the expression of the LacZ reporter gene was not detected in limb skeletal primordia (Fig. 1a–c). Around the newborn stage, β-galactosidase activity was gradually detected in the femoral diaphysis (Fig. 1d). Prominent X-gal stained signals were distributed throughout the femur (metaphysis, diaphysis and periosteum) at 3 weeks of age (Fig. 1g–j). The mono-nucleic flat cells (at the diaphysis and periosteum; Fig. 1i,j) and multi-nucleic cells (at the metaphysis; Fig. 1j) showed strong X-gal stained signals. LacZ reporter activity was observed in sFRP4 ^LacZ/+^ craniofacial and lower abdominal regions at the embryonic stage (data not shown). β-galactosidase activity was not observed in wild type mice (Supplementary Fig. 2). In 3-week-old mice, immunocolocalization of osterix confirmed that the mono-nucleic flat cells expressing β-galactosidase corresponded to osteoblastic cells (Fig. 1l). Furthermore, immunocolocalization with Cathepsin K demonstrated that X-gal stained multi-nucleic cells corresponded to osteoclastic cells (Fig. 1k).

### Bone phenotypes of sFRP4-deficient mice

The results of the histological LacZ assay led to our examining the functional roles of sFRP4 in the development of postnatal bone (Fig. 1). All sFRP4-deficient mice were born at the expected Mendelian ratio without observable defects after birth (data not shown).

For the initial analysis of the phenotype of skeletal bone, neonatal skeletons including the head, axis and limbs of sFRP4 ^LacZ/LacZ^ and their sFRP4 ^LacZ/+^ littermates were subjected to double staining with alcian blue and alizarin red. At the neonatal stage, ossification was almost complete in both sFRP4 ^LacZ/LacZ^ and sFRP4 ^LacZ/+^ skeletons (Supplementary Fig. 3).

**Figure 2:**
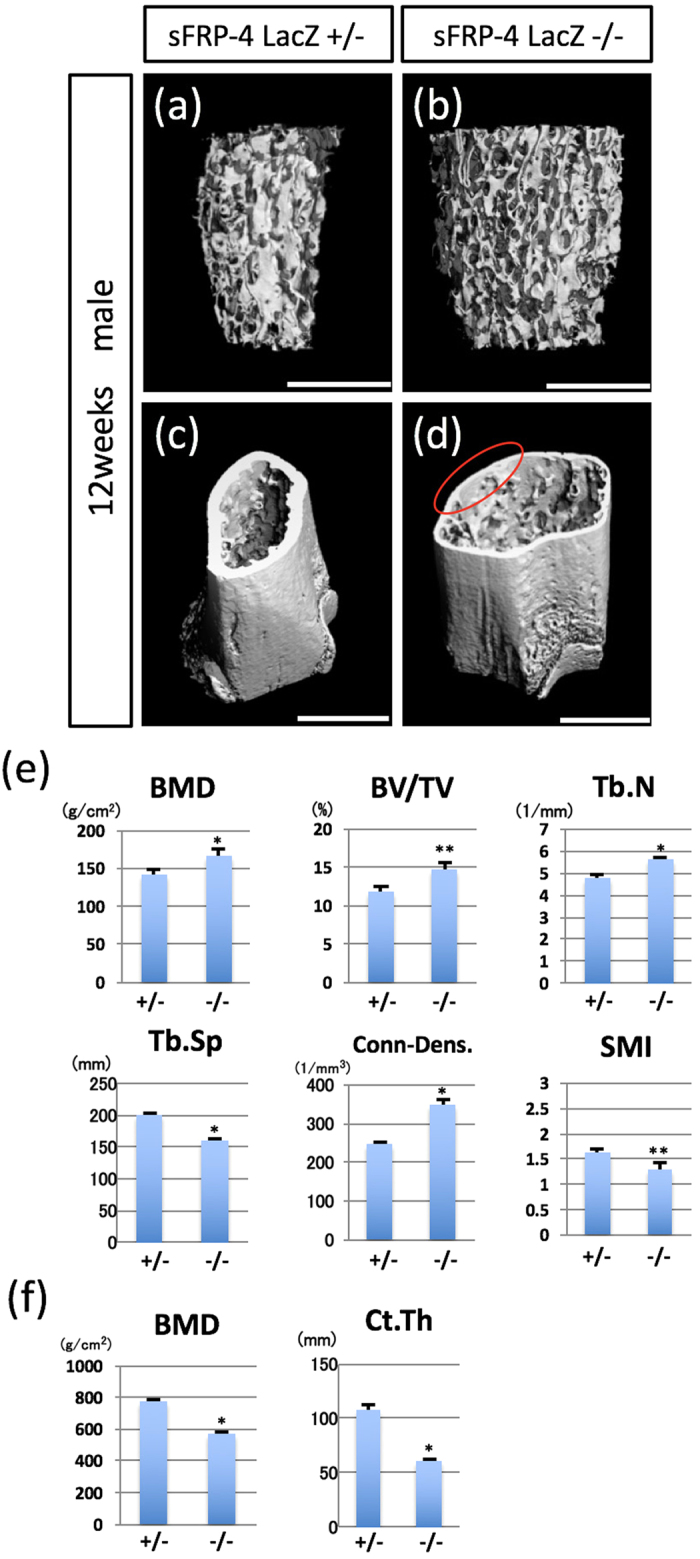
Abnormal bone formation induced by targeted inactivation of the sFRP4 gene. (**a–d**) Representative 3D μCT images of the distal femurs of sFRP4 ^LacZ/+^ and sFRP4 ^LacZ/LacZ^ mice at 12 weeks of age. μCT images of trabecular bone (**a**,**b**) and cortical/trabecular bone (**c**,**d**). Note increased trabecular mass (**b**) and reduced cortical width ((**d**) red circle) in sFRP4 ^LacZ/LacZ^ mice. (**e**) Quantification of Bone mineral density (BMD), Bone volume fraction (BV/TV), Trabecular number (Tb.N), Trabecular spacing (Tb.Sp), Connectivity density (Conn-Dens) and Structure model index (SMI) of trabecular bone of distal femurs. (**f**) Quantification of Bone mineral density (BMD) and Cortical thickness (Ct.Th) of cortical bone of distal femurs. Bone structural parameters in sFRP4 ^LacZ/LacZ^ mice show the buildup of trabecular bone and the weakening of cortical bone. Results of all quantitative analysis (**e**,**f**) are consistent with 3D μCT images (**a–d**). Error bars indicate the means ± SD (n=5); means ± SD in BMD: 40.87 ± 7.87 for +/− and 166.09 ± 8.58 for −/−, BV/TV: 11.84 ± 0.58 for +/− and 14.85 ± 0.88 for −/−, Tb.N: 4.82 ± 0.09 for +/− and 5.68 ± 0.07 for −/−, Tb.Sp: 199.33 ± 5.06 for +/− and 161 ± 2.72 for −/−, Conn-Dens: 249.04 ± 3.30 for +/− and 348.96 ± 11.48 for −/−, SMI: 1.63 ± 0.08 for +/− and 1.3 ± 0.12 for −/−, cortical BMD: 771.66 ± 13.9 for +/− and 569.03 ± 14.07 for −/−, Ct.Th: 107.47 ± 4.78 for +/− and 61.1 ± 1.42 for −/−. Statistical significance was determined by paired Student’s *t*-test. *P < 0.01 and **P < 0.05 versus sFRP4 LacZ +/−. Bars 1000 μm (**a–d**).

Neonatal mice deficient in sFRP4 showed no gross abnormalities; however, analyses of micro-computed tomography (μCT) revealed that functional loss of sFRP4 caused significant alteration of bone structures in the mid-postnatal period (Fig. 2). The femora of the 12-week male sFRP4 ^LacZ/LacZ^ mice and their sFRP4 ^LacZ/+^ littermates were also analyzed by μCT. 3D μCT images disclosed a markedly increased trabecular bone mass in the distal femur of sFRP4 ^LacZ/LacZ^ compared with that of sFRP4 ^LacZ/+^ mice (Fig. 2a,b). In contrast, cortical bone mass and thickness were decreased in sFRP4 ^LacZ/LacZ^ mice (Fig. 2c,d). These observations were statistically confirmed by quantitative μCT analysis (sFRP4 ^LacZ/LacZ^ vs. sFRP4 ^LacZ/+^). Alterations of all bone parameters, including Bone mineral density (BMD), Bone volume fraction (BV/TV), Trabecular number (Tb.N), Trabecular spacing (Tb.Sp), Connectivity density (Conn-Dens) and Structure model index (SMI), indicated structural bone augmentation in sFRP4-deficient trabecular bone (Fig. 2e). In contrast, cortical bone parameters, Bone mineral density (BMD) and Cortical thickness (Ct.Th), demonstrated structural bone degradation (Fig. 2f).

### Histological and molecular aspects of sFRP4-deficient bone

**Figure 3:**
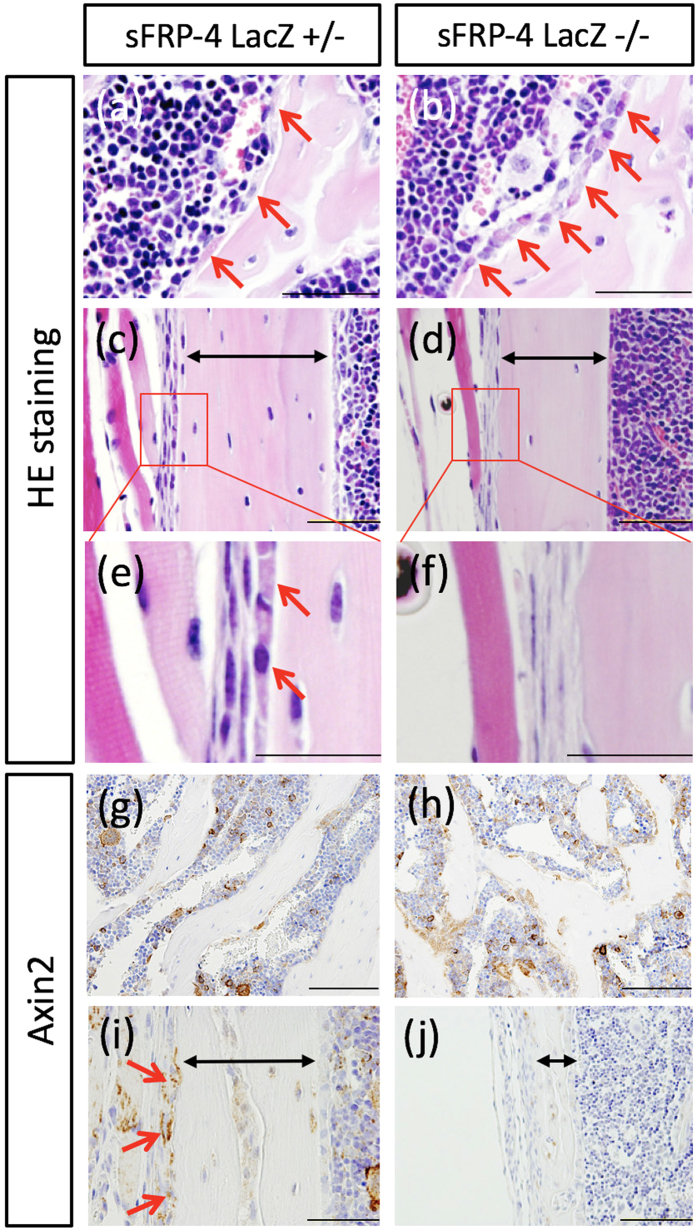
Histological examination of osteogenic cell lineages in sFRP4 ^LacZ/LacZ^ mice. Femurs from sFRP4 ^LacZ/+^ and sFRP4 ^LacZ/LacZ^ mice at 5 weeks of age were processed for histological analyses. (**a–e**) Representative images of femur stained with hematoxylin and eosin. Cuboidal cells in the trabecular bone surface are more numerous in sFRP4 ^LacZ/LacZ^ mice compared with those in sFRP4 ^LacZ/+^ mice (**a**,**b**; red arrows). Distinct flattened cells in the periosteum (**e**; red arrows) are absent in sFRP4 ^LacZ/LacZ^ mice (**f**). (**g–j**) Axin2 protein expression at the trabecula and the periosteum. (**i**,**j**) Axin2 protein expression is strikingly decreased in the sFRP4 ^LacZ/LacZ^ periosteum compared with that in sFRP4 ^LacZ/+^ mice (red arrows, signal positive cells ratio; control 12% and KO 32%). (**g**,**h**) In contrast, Axin2 protein expression in the sFRP4 ^LacZ/LacZ^ trabecular region is higher than that in sFRP4 ^LacZ/+^ (positive stained cells ratio; control 24% and KO 48%). Bidirectional arrows denote periosteal width (**c,d,i,j**). Magnifications of the red boxed regions (**c**,**d**) are shown in (**e**,**f**). Bars 50 μm (**a–d**), 25 μm (**e**,**f**), 100 μm (**g–j**).

To define the cellular phenotype of sFRP4 ^LacZ/LacZ^ skeletons, the distal femurs of sFRP4 ^LacZ/LacZ^ and their sFRP4 ^LacZ/+^ littermates at 5 weeks of age were stained with Hematoxylin and Eosin (HE) for initial histological analysis (Fig. 3a–e). Compared with that of sFRP4 ^LacZ/+^ mice, the trabecular region of sFRP4 ^LacZ/LacZ^ femurs showed numerous cuboidal cells with slightly basophilic cytoplasm representing osteoblast-like histological features rimmed with trabecular bone spicules (Fig. 3a,b). In contrast, compared with that of sFRP4 ^LacZ/+^ mice, the width of the cortical bone of sFRP4 ^LacZ/LacZ^ femurs was greatly reduced (Fig. 3c,d), and at a higher magnification, showed markedly decreased flattened cells with slightly basophilic cytoplasm in the region of the periosteum (Fig. 3e,f). sFRP4 ^LacZ/LacZ^ femur bone phenotypes were thus characterized through histological analysis (3D μCT images in Fig. 2).

**Figure 4:**
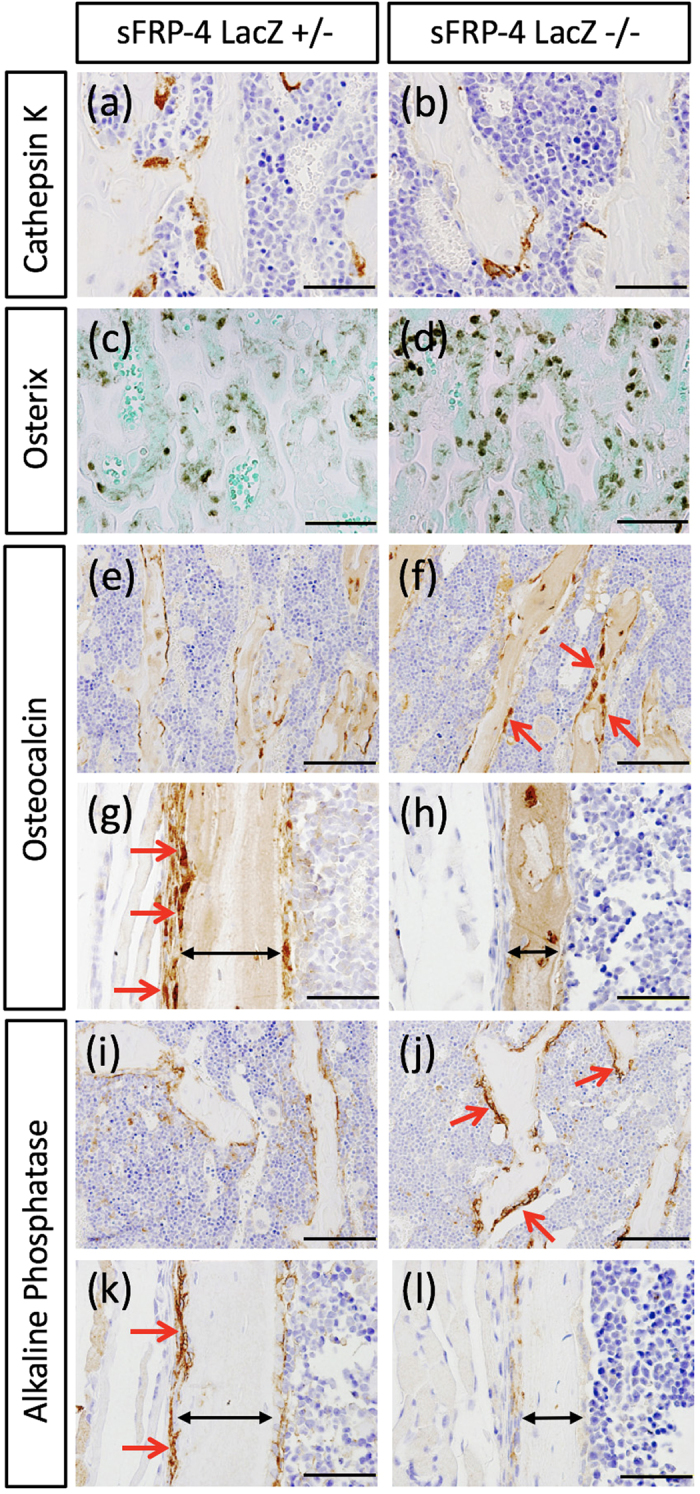
Histological molecular characterization of sFRP4 ^LacZ/LacZ^ mice. (**a,b**) A significant decrease of cathepsin K (osteoclast marker) protein expression is observed in sFRP4 ^LacZ/LacZ^ compared with that in sFRP4 ^LacZ/+^ mice (positive stained cells ratio; control 31% and KO 10%). Supplementary Fig. 4. shows results of histochemical TRAP staining of monitored osteoclast activity. (**c**,**d**) Prominent Osterix expression in the trabecular region is observed in sFRP4 ^LacZ/LacZ^ compared with that in sFRP4 ^LacZ/+^ mice (control 41% and KO 62%). (**e–l**) Expression pattern of bone deposition markers, osteocalcin and alkaline phosphatase: in the trabecular region, both markers are strongly expressed in sFRP4 ^LacZ/LacZ^ compared with those in sFRP4 ^LacZ/+^ mice (osteocalcin; control 28% and KO 55%, alkaline phosphatase; control 30% and KO 53%); in the periosteal region, the expression of both markers is significantly repressed (**g,h,k,l**; red arrows, osteocalcin; control 39% and KO no-clear signal, alkaline phosphatase; control 79% and KO 16%). Bidirectional arrows denote periosteal width (**g,h,k,l**). Bars 50 μm (**a,b,e,f,i,j**), 100 μm (**c,d,g,h,k,l**).

Next, to examine whether these bone phenotypes of sFRP4 ^LacZ/LacZ^ mice are elicited in response to changes in Wnt signaling activity, the expression pattern of Axin2, the direct target gene of the Wnt signaling pathway^24^, was investigated by immunohistochemistry (Fig. 3g–j). In sFRP4 ^LacZ/+^ trabecular regions, Axin2 immunostaining signals were detected in the cytoplasm of mono- and multi-nucleic cells rimmed with bone spicules (Fig. 3g), whereas the signals were augmented in sFRP4 ^LacZ/LacZ^ mice (Fig. 3h). That Axin2, β-catenin and LEF1 mRNA levels were elevated in sFRP4 ^LacZ/LacZ^ primary osteoblasts was also verified through qRT-PCR analysis (Supplementary Fig. 5c). In contrast, cortical Axin2 immunostaining signals were detectable in only periosteal cells of sFRP4 ^LacZ/+^ mice (Fig. 3i); the signals were totally absent from sFRP4 ^LacZ/LacZ^ specimens (Fig. 3j). We next asked and investigated whether this altered Wnt signal activity affected the expression of osteogenic and bone deposition molecular markers (Fig. 4a–l). Cathepsin K, the mature osteoclast marker^25^, was detected in the trabecular regions of sFRP4 ^LacZ/+^ femurs, whereas it decreased markedly in sFRP4 ^LacZ/LacZ^ specimens (Fig. 4a,b). In addition, the suppressed activity of osteoclasts was confirmed by histochemical staining with TRAP (Supplementary Fig. 4). Osterix expression, one of the osteoblast progenitor markers^26^,^27^, was elevated in sFRP4 ^LacZ/LacZ^ trabecular regions compared with that in sFRP4 ^LacZ/+^ (Fig. 4c,d). The expression of common bone deposition marker genes, osteocalcin and alkaline phosphatase^6^,^28^, was also dramatically changed in sFRP4 ^LacZ/LacZ^ femurs. Both markers were strongly expressed in the trabecular region of sFRP4 ^LacZ/LacZ^ (Fig. 4e,i,j) and strongly repressed in the periosteal region (Fig. 4g,h,k,l). In addition, the effects of sFRP4-inactivation on bone formation and resorption markers were examined by qRT-PCR analysis (Supplementary Fig. 5a,b). Previous studies have shown that activated Wnt signaling promotes bone formation by stimulating several osetogenic genes^29^,^30^,^31^, and suppresses bone resorption by direct negative influence on osteoclastogenesis^32^,^33^. These histological observations were consistent with Wnt signal activity modulations represented by the expression of Axin2 in sFRP4 ^LacZ/LacZ^ (Fig. 3g–j and Supplementary Fig. 5c).

### Bone phenotypes of aged sFRP4-deficient mice

**Figure 5:**
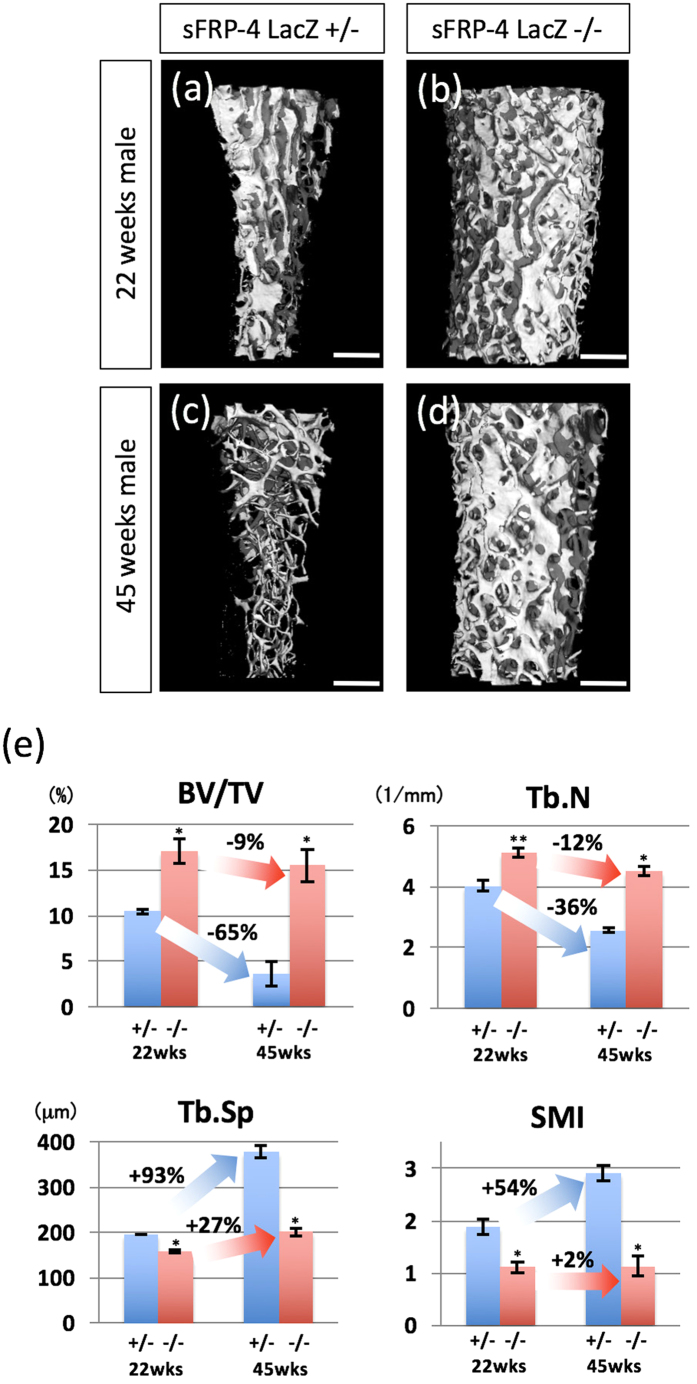
sFRP4 gene inactivation prevents age-related bone loss. (**a–d**) Representative 3D μCT trabecular bone images of the distal femur of control and sFRP4 ^LacZ/LacZ^ mice at 22 and 45 weeks of age. In control mice, note greatly reduced trabecular mass indicative of trabecular osteopenia at 45 weeks (**c**) compared with that at 22 weeks (**a**). sFRP4 gene inactivation strikingly prevents age-related trabecular osteopenia in sFRP-4LacZ−/− (**d**) compared with control mice (**c**), at 45 weeks. (**e**) Quantification of Bone volume fraction (BV/TV), Trabecular number (Tb.N), Trabecular spacing (Tb.Sp) and Structure model index (SMI) of trabecular bone at distal femurs. Compared with control mice, sFRP4 ^LacZ/LacZ^ mice show no significant age-related differences (22 weeks vs. 45 weeks) in all structural bone parameters. The degree of variability (%) is shown in bar graphs. Error bars indicate the means  ±  SD (n=5); means ± SD in BV/TV: 10.44 ± 0.28 for 22wks+/−, 17.11 ± 1.32 for 22wks−/−, 3.6 ± 0.97 for 45wks+/− and 15.51 ± 1.8 for 45wks−/−, Tb.N: 4.03 ± 0.18 for 22wks+/−, 5.12 ± 0.16 for 22wks−/−, 2.57 ± 0.1 for 45wks+/− and 4.52 ± 0.14 for 45wks−/−, Tb.Sp: 196.37 ± 0.47 for 22wks+/−, 158.23 ± 2.1 for 22wks−/−, 378.97 ± 13.28 for 45wks+/− and 201 ± 7.78 for 45wks−/−, SMI: 1.88 ± 0.15 for 22wks+/−, 1.11 ± 0.11 for 22wks−/−, 2.91 ± 0.15 for 45wks+/− and 1.14 ± 0.2 for 45wks−/−. Statistical significance was determined by paired Student’s *t*-test. *P < 0.01 and **P < 0.05 versus sFRP4 LacZ +/−. Bars 900 μm (**a–d**).

Bone loss under aging and oxidative stress is related to the reactivation of sFRP4 gene expression^20^,^21^,^34^. To address the physiological role of sFRP4 in the ageing process, we examined, with the use of μCT, the bone phenotype of sFRP4 ^LacZ/LacZ^ and male control mice at 22 and 45 weeks of age (Fig. 5). The femur of male control mice at 45 weeks of age, compared with that at 22 weeks (age at peak bone mass in mice^35^,^36^), displayed extremely scant trabeculae because of age-dependent bone loss (Fig. 5a,c). Interestingly, sFRP4 ^LacZ/LacZ^ at 45 weeks of age retained much of the trabecular bone as at 22 weeks of age (Fig. 5b,d); also, the cortical bone phenotype demonstrated no significant age-dependent bone loss between these two age groups (data not shown); these bone phenotypes were characterized by histological analysis (Supplementary Fig. 6). We also examined the serum markers of bone turnover by ELISA (Supplementary Fig. 7). Serum osteocalcin, a marker of bone formation, was significantly higher in sFRP4 ^LacZ/LacZ^ compared with that in control mice at 45 weeks. In contrast, serum concentrations of Trap5b showed no significant difference. Next, to better define these bone phenotypes, distal femurs were analyzed by quantitative μCT (Fig. 5e). Mice achieved peak in several bone parameters at around 20 weeks of age; thereafter, bone parameters degraded gradually and significantly^35^,^36^ (data not shown). Compared with trabecular bone parameters of 22-week-old mice, BV/TB was 65% lower, Tb.N 36% lower, Tb.Sp 93% higher, and SMI 54% higher in 45-week-old mice (these parameter changes signified embrittlement of bone structure; bold blue arrows in Fig. 5e). Compared with control mice, sFRP4 ^LacZ/LacZ^ mice surprisingly prevented natural age-associated bone loss at 45 weeks of age. In sFRP4 ^LacZ/LacZ^ mice at 45 weeks, BV/TB was 9% lower, Tb.N 12% lower, Tb.Sp 27% higher, and SMI 2% higher as compared with 22-week-old mice (bold red arrows in Fig. 5e). sFRP4 ^LacZ/LacZ^ female mice at 45 weeks of age also exhibited age-dependent phenotypes similar to those of male mice (Supplementary Fig. 8). Thus, the data of quantitative μCT analysis showed that the fluctuation margin in bone parameters of sFRP4 ^LacZ/LacZ^ mice between 22 and 45 weeks of age was insignificant, compared with that in control mice.

## Discussion

The sFRP4 gene is a negative regulator of the Wnt signaling pathway that promotes the buildup of bone strength and bone mass^20^,^21^. Excess amounts of sFRP4 are involved in bone loss attributed to osteoblastic inactivation during aging and oxidative stress^20^,^21^,^34^. To explore the physiological characteristics of sFRP4 in bone development and homeostasis, we engineered an sFRP4 ^LacZ^ *knock-in* mouse strain. Analysis of this strain demonstrated that the sFRP4-mediated Wnt signal modulation system has a deep impact on postnatal skeletal development and adult bone maintenance. Analyses of the functional characteristics of sFRP4 LacZ reporter *knock-in* mice revealed the following: (1) after birth, sFRP4 is gradually expressed in cells of both osteoblastic and osteoclastic cell lineages; (2) sFRP4-deficient trabecular bone has a high bone mass, whereas that deficiency in cortical bone shows reduced thickness; (3) sFRP4-deficient trabecular bone phenotypes show resistance to natural age-associated bone loss through enhanced Wnt signaling activity. Based on these findings, we speculate that sFRP4 plays an essential role in both osteogenic cell formation/differentiation (osteoblasts and osteoclasts) during postnatal bone development and bone remodeling processes.

### Unique expression of sFRP4 gene in skeletal bone tissues

**Figure 6:**
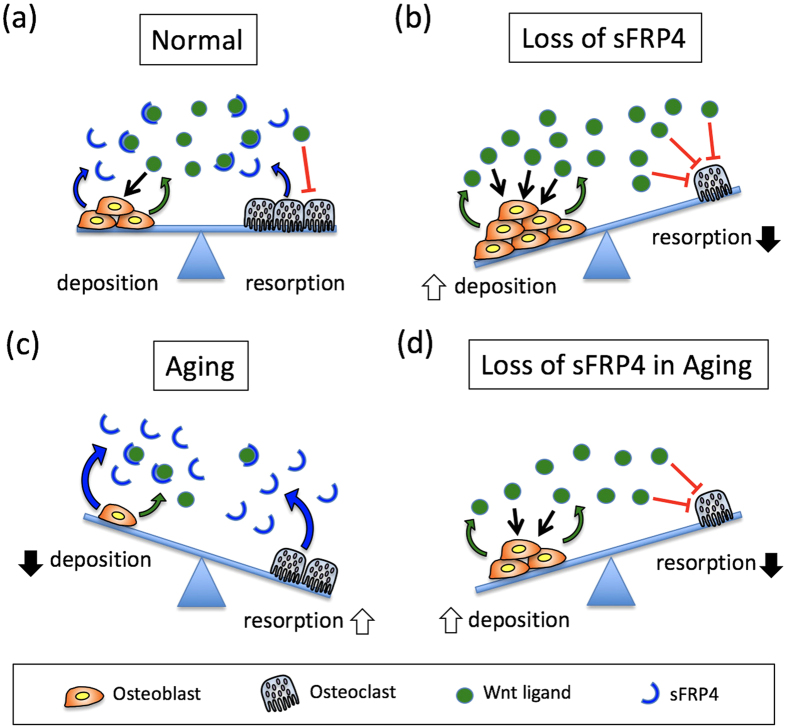
(**a–d**) Contribution of sFRP4 to bone remodeling. Wnt signals derived from osteoblasts (bold green arrows) promote osteoblast proliferation and differentiation (arrows indicate positive effects) and repress osteoclast formation (red T-shaped bars indicate inhibitory effects). sFRP4 produced in osteoblasts and osteoclasts (bold blue arrows) fine-tunes the balance between bone deposition and resorption through the functional blocking of Wnt ligands. (**a**) Bone remodeling is controlled by balanced amounts of Wnt ligands and sFRP4. (**b**) Functional loss of sFRP4 causes a synergistic enhancement of bone deposition by increasing osteoblast activity and repressing osteoclast formation. (**c**) Bone resorption develops as a result of reduced osteoblastic expression of Wnt ligands and elevated sFRP4 expression that blocks the inhibitory effect of Wnt signal on osteoclast formation. (**d**) Functional loss of sFRP4 in aged bone (“Loss of sFRP4 in Aging”) prevents age-related bone loss by recovering the Wnt signal activity for osteoblast and osteoclast formation.

Studies on sFRP4 gene transcription in skeletal tissues of several animal species, based mainly on *in situ* hybridization and quantitative PCR methods, have shown that sFRP4 is prominently expressed in skeletal regions during late embryonic and postnatal development stages^21^,^22^,^23^,^34^. Our histological examination of sFRP4-LacZ *knock-in* mice, a genetic approach using a highly sensitive reporter system^37^, assigned, for the first time, sFRP4-expressed cells to two distinct cell types in postnatal bone tissues (Fig. 1). After birth, sFRP4 endogenous promoter-driven LacZ expression was gradually localized at mono- and multi-nucleated cells in metaphysial, diaphysial and periosteal regions, and combined X-gal and IHC staining revealed that the majority of these LacZ-expressed cells contained both osteoblastic and osteoclastic lineage cells. For the significance and relevance of Wnt signaling to osteogenesis, while some studies have shown that the activated-Wnt signal by Wnt ligands derived from osteoblastic lineage cells regulates their formation and differentiation^38^, others have demonstrated that the activated-Wnt signal on osteoclasts has a direct negative effect on their formation by *in vitro* cell culture assays^33^ and by phenotypic analysis of the myeloid cell lineage^32^ in β-catenin-deficient mice. Indeed, Wnt16 derived from osteoblasts inhibits osteoclastogenesis by acting as an osteoblast-osteoclast coupling factor on osteoclasts^39^. Our current precise expression profile of the sFRP4 gene also illustrates the intriguing possibility that sFRP4, expressing both cell types, is a new potential endogenous modulator of the Wnt signaling pathway for the osteoblast-osteoclast coupling process (Fig. 6).

### Regulative role of sFRP4-mediated Wnt signaling modulation on the bone formation and bone resorption

The expression and function of WNT antagonists has been of interest in the regulation of bone formation^29^. Master osteogenic regulator Runx2, that controls the expression of mature osteoblast marker genes like osteocalcin, is directly regulated by the canonical WNT signaling pathway; the Runx2 gene promoter has a regulatory element that directly interacts with Wnt signaling components in osteoblasts^40^. In this study, we showed that the expressions of Runx2 and osteocalcin were strikingly up-regulated in sFRP4-deficiet primary osteoblasts (Supplementary Fig. 5a). Our data suggest that WNT signaling activation by the loss of sFRP4-mediated Wnt antagonism promotes enhancement of bone formation with elevation of Runx2, thus contributing to osteoblast differentiation. As in osteogenesis, sFRP4 bone phenotype analyses *suggested* that inhibition of sFRP4-dependent Wnt signaling modulation directly *affected* osteoclastogenesis; sFRP4 *was* expressed in osteoclasts, and bone resorption *decreased* in sFRP4-deficient trabeculae with the suppression of the expression of osteoclastic markers, cathepsin K and TRAP. The direct influence of the canonical WNT signaling pathway to osteoclastogenesis has been described^32^,^33^. Myeloid lineage specific β-catenin-deficient mice display increased bone resorption with the promotion of osteoclastogenesis^32^, and administration of Wnt3a, one of the ligands of the canonical Wnt signaling pathway, shows strong anti-osteoclastogenic effect on the bone marrow cell-culture system^32^,^33^. In sFRP4-deficient trabeculae, Wnt-signal-activated osteoclasts (Cathepsin K and Axin2 double positive cells) were increased compared with those in the control (data not shown). We therefore consider that sFRP4-dependent Wnt signaling modulation is one of the key regulatory mechanisms that directly affects osteoclastogenesis.

### Phenotypic difference between trabecular and cortical bone in sFRP4 deficient mice

In addition to the role of sFRP4 in skeletal bone formation during mouse developmental periods (Fig. 2), its loss resulted in a phenotype of increased trabecular bone mass attributed to both excessive bone deposition and suppression of bone resorption, the cellular phenotypes of which were confirmed by the analysis of several osteogenic markers: Osterix, Cathepsin K and TRAP (Fig. 4 and Supplementary Fig. 4). The expression pattern of Axin2, a direct downstream gene of Wnt signaling, also confirmed that an osteogenic cellular response to Wnt signaling modulated the osteogenic phenotype in trabecular bone. Consistent with our findings, previous genetic mutational studies have shown that the gain-of-function mutation in LRP5 and Wnt10b and the loss-of-function mutation in sFRP1 induce changes in structural bone, resulting in a phenotype of increased bone mass in the trabecular region, with enhancement of osteoblastic activity attributed to the activated-Wnt signaling pathway^7^,^41^,^42^.

There is, however, a significant phenotypic difference between sFRP4-deficient mice and a series of gain-of-function mutants in Wnt signaling. In contrast to functional mutants LRP5, Wnt10b and sFRP1 that show phenotypes of increased bone mass, sFRP4-deficient mice exhibit significant reduction in cortical bone compartments (Fig. 2). Moreover, our data show that Axin2 expression decreased significantly in the sFRP4-deficient cortical region, contrary to the predicted lack of Wnt antagonism by sFRP4 targeted null-mutation (Fig. 3). Indeed, most sFRPs function as Wnt signaling antagonists by direct binding to Wnt ligands^13^; they can also modulate the activity of other signaling pathways^43^,^44^,^45^ by direct physical interaction with molecules unrelated to multiple Wnt ligands^14^ such as fibronectin, receptor activator for nuclear factor κB ligand (RANKL) and BMP1/Tolloid, a metalloprotease that promotes BMP signaling. We therefore speculate that sFRP4 can function as one of the key molecules that determine the axis between cortical and trabecular bone mass by modulating the fate of osteoblastic cell lineages through the interaction of yet-unrecognized target molecules that exert a negative influence on Wnt signaling solely in the cortical bone region.

### Functional loss of sFRP4 leads to dramatic prevention of natural age-associated bone loss

While we discovered that functional loss of sFRP4 gives rise to increased trabecular bone mass with the synergistic enhancement of bone formation through the activation of osteoblasts and the inactivation of osteoclasts, we also demonstrated that this augmented effect of osteogenesis maintains bone mass and prevents age-dependent bone loss in aged mice (Fig. 5). Activation of the Wnt signaling pathway through the loss of sFRP4 function has beneficial effects on aged bone^21^. Indeed, phenotypic observations of LRP5 and Wnt10b gain-of-function and sFRP1 loss-of-function have revealed that adequate Wnt signaling regulation is a critical element for not only skeletal bone formation but also bone remodeling throughout life^7^,^41^,^42^. Evidence of the importance of sFRP4-mediated Wnt signal modulation in bone physiology has demonstrated that sFRP4 can be age-dependently up-regulated in a Senescence-accelerated mouse strain (SAMP6)^34^ (a human model of age-related bone loss) and that functional activation of this gene in the osteoblastic cell lineage results in a decrease of trabecular bone mass, which phenotype is opposite to that of sFRP4 ^LacZ/LacZ^ mutants that inextricably associate with each other^5^. Moreover, our previous studies have shown that among sFRPs, sFRP4 expression is selectively activated under continual oxidative stress such as that of aging, which leads to low bone mass because of osteoblastic inactivation by suppressed Wnt signaling^20^,^21^,^34^. In contrast to other sFRP gene families that contain numerous CpG loci that form typical CpG-islands, the basic promoter region of the sFRP4 gene locus contains scattered CpG-loci^20^. When methylated, sFRP4 gene silencing is achieved by MeCP2 protein binding at its typical site (cgcgtctggataaata) located adjacent to TATA-box, then oxidative stress rapidly derepresses the epigenetically silenced sFRP4 gene by sequestering MeCP2 while keeping methylated cytosine unchanged^20^. Thus, after sFRP gene silencing by age-dependent promoter methylation, highlighted was the intriguing possibility that of these, reactivated sFRP4 plays the central role in age-related bone loss as the only potential endogenous modulator of the Wnt signaling pathway for the bone remodeling process in response to oxidative stress or aging (Fig. 6).

To summarize, this study of sFRP4-LacZ *knock-in* mice revealed that sFRP4 affects the physiology of both osteoblasts and osteoclasts by modulating the Wnt signaling pathway. Loss of sFRP4 leads to the promotion of osteo-progenitor proliferation/differentiation and the suppression of osteoclastic activity. These mechanisms synergistically enhance bone deposition during mouse developmental and remodeling processes of aging. Effective bone therapeutic drugs that enhance both promotion of osteogenesis and prevention of osteolysis are currently unknown^46^,^47^. Accordingly, by providing a detailed understanding of how sFRP4-mediated Wnt signaling contributes to bone biology (for example, characterization of the crystal structure or *in silico* design of specific peptide inhibitors of sFRP4), our findings could have future clinical implications for the development of novel drugs effective in the treatment of age-related bone loss.

## Methods

### sFRP4 LacZ reporter knock-in mice

The knock-in schematic is shown in Supplementary Fig. 1. A targeting vector was designed to produce a null-allele mutation of sFRP4 by replacing the first exon including the start codon (ATG) with a nlacZ-neo selection cassette (Supplementary Fig. 1a). The sFRP4 mutant mice (Accession No. CDB0587K: http://www2.clst.riken.jp/arg/mutant%20mice%20list.html) were then established as described (http://www2.clst.riken.jp/arg/Methods.html). The PCR assay used to genotype the mice was conducted with the use of purified DNA from tail lysates with the following primers (Supplementary Fig. 1a): 5′-TCCTCATCAGTGCAGACTGG-3′ (P1), 5′-GCAGGAACTCCAGGGTACAG-3′ (P2), and 5′-GATTCTCCGTGGGAACAAAC-3′ (P3). All animal experimental procedures and protocols were approved by the Committee on Animal Research at Ehime University (Permit Number: 05-KU-24-16) and Institutional Animal Care and Use Committee of RIKEN Kobe Branch (Permit Number: AH13-03-65); the experiments were carried out in accordance with the approved guidelines. Mice were sacrificed by cervical dislocation.

### X-gal staining in skeletons

LacZ reporter gene expression was detected as previously described with the following modifications. Dissected adult bones were fixed in 0.8% paraformaldehyde (PFA) and 0.02% glutaraldehyde in PBS for 2days at 4 °C and washed five times in PBS. The bones were decalcified in 20% EDTA for 5 days and then washed three times in PBS for 1 h before X-gal staining by standard procedures 37,48. The bones were washed once with PBS and then fixed in 4% PFA at 4 °C overnight. Individual bones were rinsed in 70–100% ethanol, embedded in paraffin wax, sectioned at 6 μm, and counterstained with eosin. Neonatal and embryonic skeletons were not decalcified before X-gal staining.

### Histological analysis

Postnatal and adult mouse femurs were dissected and fixed in 4% PFA/PBS for 2 days at 4 °C. Bone tissues were decalcified in 20%EDTA for 5days. Embryonic and neonatal tissues were fixed overnight in 4%PFA/PBS, but not decalcified. Fixed skeletal tissues were dehydrated through ethanol and embedded in paraffin; 8-μm serial sections were then prepared for histological analysis^49^. Hematoxylin and Eosin (HE) staining and immunohistochemical analyses were carried out by standard procedures using the following antibodies (Ab)^50^: anti-Cathepsin K (1:300, cat. no. ab19027, abcam)^51^, anti-Osterix (1:1000, cat. no. ab22552, abcam)^52^, anti-Axin2 (1:300, cat. no. ab32197, abcam)^53^, anti-Osteocalcin (1:1000, cat. no. M173, Takara)^54^, anti-Alkaline Phosphatase (1:500, cat. no. M190, Takara)^55^. Bone-tissue cells with positive immunohistochemical signals representing equal areas of control and sFRP4 KO mice were counted and their average numbers were compared.

### Staining and visualization of whole skeletons

Mouse embryos were fixed for 48 hours at 4 °C in 4%PFA/PBS and for 48 hours in 95% ethanol, stained with Alcian blue for 48 hours by incubation in 0.03% Alcian blue (Muto chemicals) dissolved in 70% ethanol/20% acetic acid. After washing in 95% ethanol for five days, the embryos were stained with 0.1% Alizarin red S (Sigma) in 1% KOH for 48 hours, washed in 95% ethanol, cleared in 1% KOH and taken through graded steps into and stored in 60% glycerol.

### Analysis of bone phenotype by μCT system

After fixation with 70% ethanol, femora were scanned with a Scanco Medical μCT35 System (SCANCO Medical) to measure bone structural parameters according to the manufacturer’s instructions and the recent guidelines of the American Society for Bone and Mineral Research (ASBMR)^56^. μCT analyses were conducted as described^57^. Image reconstruction was done on 6-μm voxels. The following bone structural parameters were calculated and statistically analyzed by μCT: Bone mineral density (BMD, g/cm^2^), Bone volume fraction (BV/TV, %), Trabecular number (Tb.N, 1/mm), Trabecular spacing (Tb.Sp, mm), Cortical thickness (Ct.Th, mm), Connectivity density (Conn-Dens, 1/mm^3^) and Structure model index (SMI).

### Enzyme-linked Immunoassays

The serum levels of osteocalcin and Trap5b were measured by Enzyme-linked Immunoassays (ELISAs) that were conducted according to the protocols of the Mouse Gla-Osteocalcin High Sensitive EIA Kit (Takara Bio, Shiga, Japan) for osteocalcin, and Mouse TRAP^TM^ Assay (Immunodiagnostic Systems, Tyne, UK) for Trap5b. The absorbance at 450nm was determined with the use of a plate reader: Flex Station 96ED (Molecular Devices, CA, USA). Mouse serum was collected by cardiac puncture.

### RNA extraction and RT-qPCR

Total RNA was isolated from the primary osteoblasts and pulverized long bone (marrow-free trabecular bones) with the use of an RNeasy Mini kit (Qiagen KK, Tokyo, Japan) according to the manufacturer’s instructions. Primary osteoblasts were isolated from calvariae of neonates. Briefly, tissue from each dissected calvaria was digested with 0.05% trypsin/1 mM EDTA (Wako, Osaka, Japan), incubated for 20 min at 37 °C, then Dulbecco’s modified Eagle’s medium with 15% fetal bovine serum was added to inactivate the trypsin. Digested cells were incubated for 24 h at 37 °C, and the adhering cells were used as primary osteoblasts. To assess the mRNA expression of Osterix, Runx2, osteocalcin, TRAP, cathepsin K, Axin2, β-catenin, LEF1 and glyceraldehyde-3-phosphate dehydrogenase (GAPDH), 1 mg of total RNA was reverse-transcribed to synthesize cDNA that was then amplified and quantified by the ABI PRISM 7300 Real Time PCR system (Applied Biosystems, Foster City, CA) with a use of sets of primers and probes (assay ID; Osterix, Mm03413826_mH; Runx2, Mm00501578_m1; osteocalcin, Mm04209856_m1; TRAP, Mm00475698_m1; cathepsin K, Mm00484039_m1; Axin2, Mm00443610_m1; β-catenin, Mm00483033_m1; LEF1 and GAPDH, No. 430813). mRNA expression was quantified relative to that of GAPDH in each reaction, according to the manufacturer’s protocol.

### Statistical analysis

Statistical analyses of bone were carried out by Student’s t-test to determine the significance between groups. *P* < 0.05 was considered statistically significant. All results are expressed as means ± SD.

## Additional Information

**How to cite this article**: Haraguchi, R. *et al*. sFRP4-dependent Wnt signal modulation is critical for bone remodeling during postnatal development and age-related bone loss. *Sci. Rep*. **6**, 25198; doi: 10.1038/srep25198 (2016).

## Supplementary Material

Supplementary Information
