# Supplementary material for: sFRP4-dependent Wnt signal modulation is critical for bone remodeling during postnatal development and age-related bone loss
Source: Sci Rep. 2016 Apr 27;6:25198. doi: 10.1038/srep25198 (PMC4846872; doi:10.1038/srep25198)
Supplement: Supplementary Information [file srep25198-s1.pdf]

Supplementary Information

sFRP4-dependent Wnt signal modulation is critical for bone remodeling during postnatal development and age-related bone loss

Ryuma Haraguchi, Riko Kitazawa, Kiyoshi Mori, Ryosuke Tachibana, Hiroshi Kiyonari, Yuuki Imai, Takaya Abe, Sohei Kitazawa

Supplementary Figure 1. Generation of sFRP4 knock-in mice.
Supplementary Figure 2. X-gal stained histological sections of wild-type and sFRP4-LacZ heterozygous mice (related to Figure 1).
Supplementary Figure 3. Skeletal staining with Alcian blue and Alizarin red (related to Figure 2).
Supplementary Figure 4. TRAP stained histological sections of control and sFRP4-LacZ homozygous mice (related to Figure 4).
Supplementary Figure 5. Relative quantification of the expression of osteogenic and Wnt signaling component genes (related to Figures 3 and 4).
Supplementary Figure 6. Hematoxylin and Eosin stained histological sections of control and sFRP4-LacZ homozygous mice (related to Figure 5).
Supplementary Figure 7. Quantification of the Serum markers of bone turnover (related to Figure 5).
Supplementary Figure 8. μCT images of control and sFRP4-LacZ homozygous femurs of aged female mice (related to Figure 5).

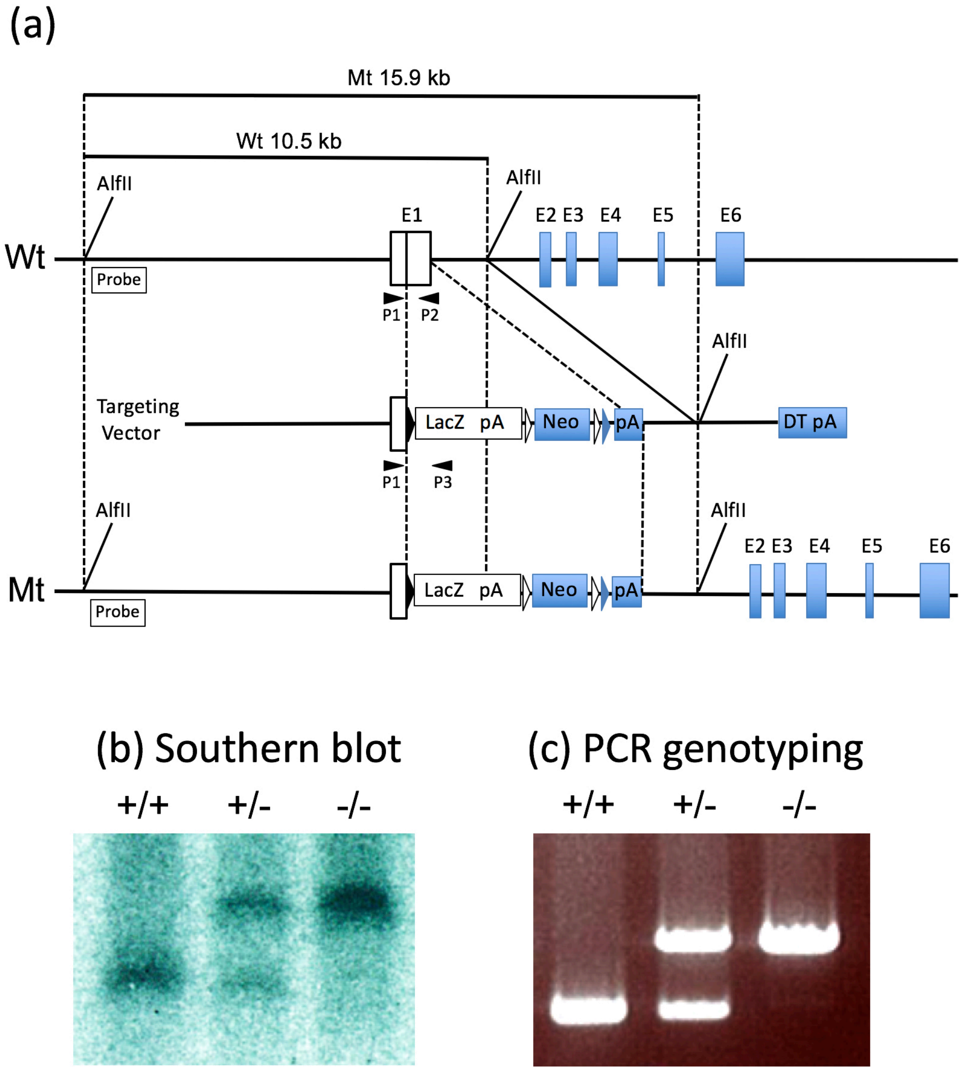

Supplementary Fig. 1. Generation of sFRP4 knock-in mice.

(a) Schematic representation of the wild-type (Wt) sFRP4 allele, targeting vector and homologous recombinant (Mt) allele. Exon1 (E1) of the mouse sFRP4 gene is replaced by LacZ and Neo selection cassette. A probe used in Southern blot analysis (shown in (b)) to detect the correct homologous recombination is shown as a boxed letter in (a). Sites of AlfII were altered by the insertion of the selection cassette and used in conjunction with sites beyond the crossing-over region to confirm homologous integration. The upper and lower bands indicate fragments corresponding to the wild-type (WT) allele and the LacZ allele, respectively. (c) Genotyping of littermate mice by genomic PCR. The shorter and longer PCR products are derived from the WT allele and LacZ allele, respectively. The primers used in genotyping PCR are shown as black arrows in (a). pA, poly-adenylation tail; DT, diphtheria toxin A fragment gene; NEO, neomycin resistance gene.

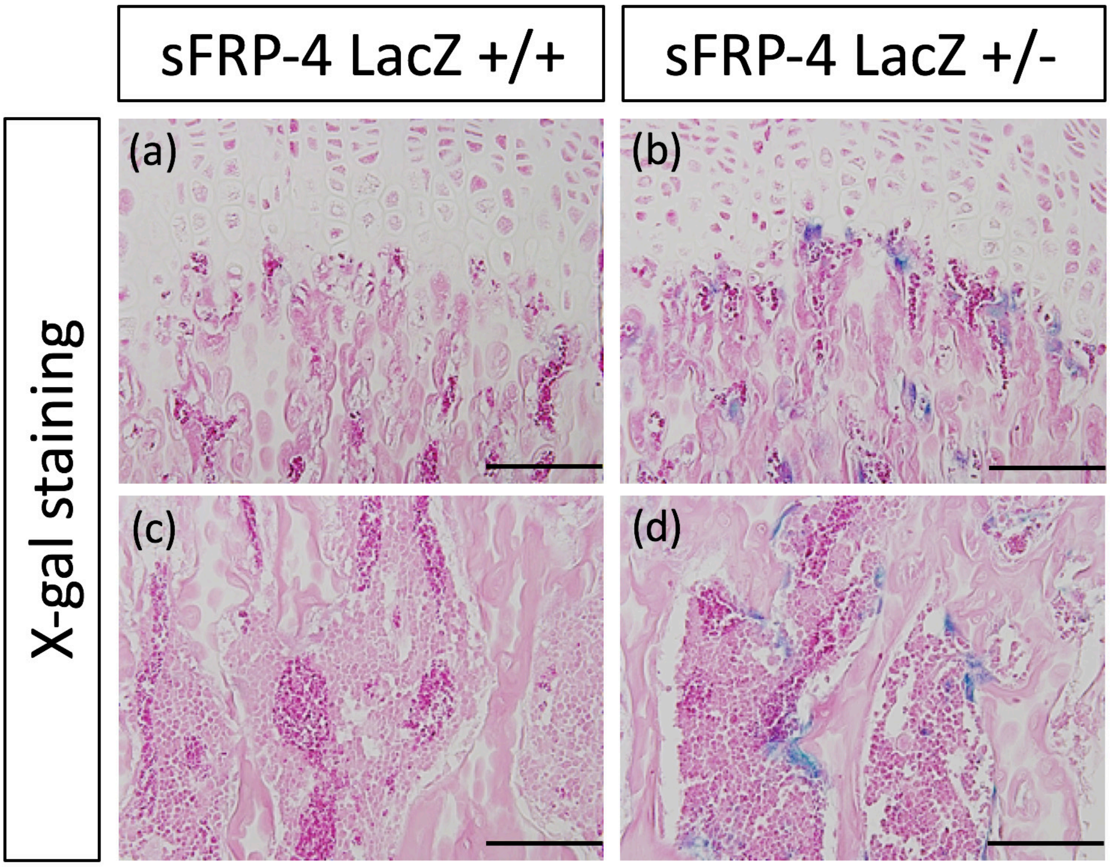

Supplementary Fig. 2. Control experiments for X-gal staining analysis, related to Figure 1

Sections of X-gal staining of the distal femur of sFRP4-LacZ wild-type (a, c) and heterozygous mice (b, d) at 3 weeks of age. Note the β-galactosidase activity in the metaphyseal (a, b) and trabecular regions (c, d). X-gal signal is not detected in wild-type (a, c). Bars 100μm

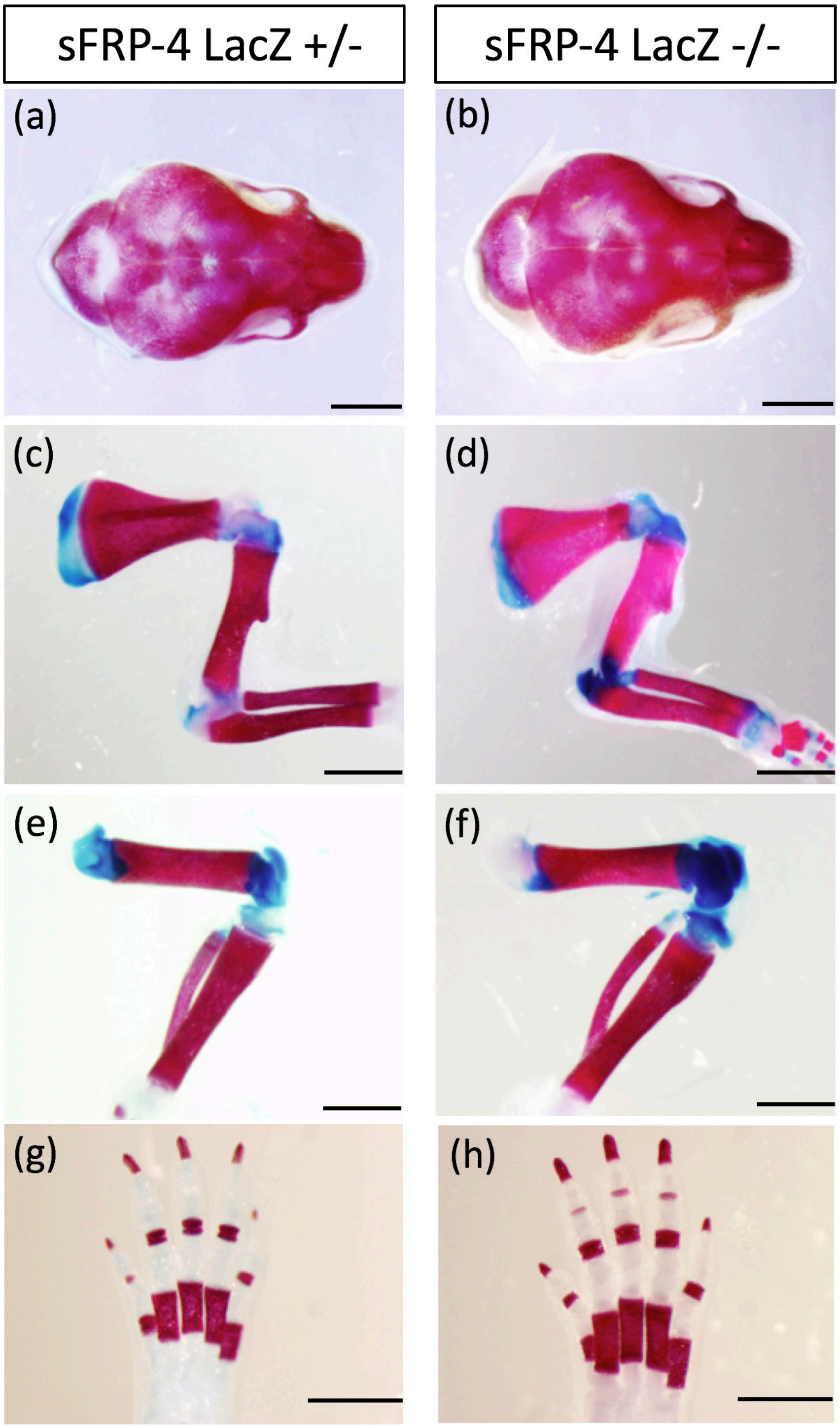

Supplementary Fig. 3. Estimation of skeletal genesis in the newborn with Alcian blue and Alizarin red staining, related to Figure 2

All images are whole-mount skeletal preparations. Skull (a, b), scapula, humerus, radius, and ulna (c, d), femur and tibia (e, f), forelimb digits (g, h) of sFRP4-LacZ heterozygous and sFRP4-LacZ homozygous mice. (b, d, f, h) sFRP4 mutants exhibit no skeletal abnormalities. Bars 2000μm (a-f), 1000μm (g, h)

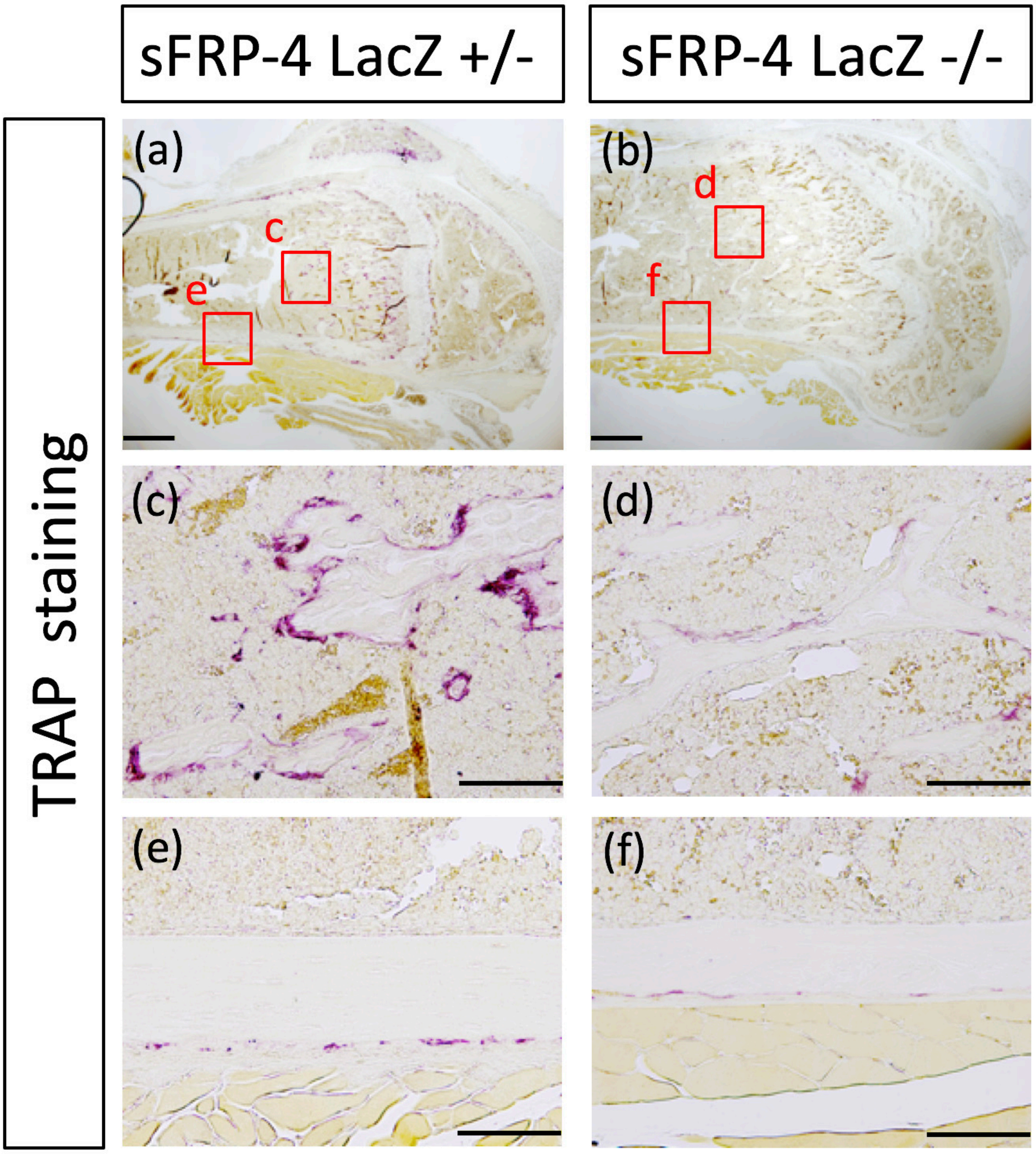

Supplementary Fig. 4. TRAP staining of sFRP4-LacZ heterozygous and homozygous femurs of 5-week-old mice, related to Figure 4

Note TRAP activity in the trabecular (*c, d*) and cortical regions (*e, f*). Signals of TRAP staining are strongly repressed in the trabeculae of homozygous mice (d). Signals of cortical TRAP staining are slightly repressed in homozygous mice (f). Magnification of the red boxed regions (a, b) are shown in (c-f). Bars 500μm (a, b), 50μm (c-h)

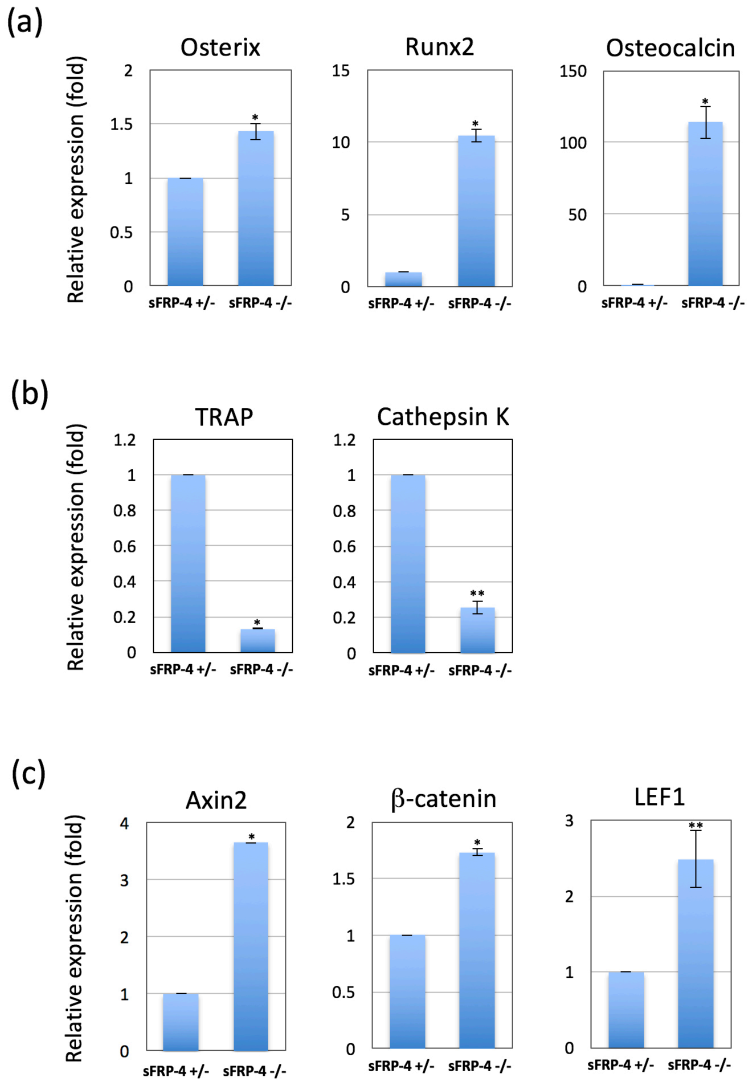

Supplementary Fig. 5. Quantitative estimation of the effect of sFRP4 inactivation on the osteogenic, bone resorptive and Wnt signaling component gene expression, related to Figure 3 and 4

(a-c) qRT-PCR expression analysis for the indicated genes. (a) Osteogenic marker genes, Osterix, Runx2 and osteocalcin, in the primary osteoblasts of control and sFRP4-LacZ -/-. (b) Bone resorption marker genes, TRAP and cathepsin K, in the trabecular bones of 5-weeks-old control and sFRP4-LacZ -/- mice. (c) Wnt signaling components, Axin2, β-catenin and LEF1, in the primary osteoblasts. See "Methods" for RNA preparation and the transcript levels normalized to GAPDH values. Error bars indicate the means ± SD (n=3); means±SD in Osterix: 1.43±0.01 for -/-, Runx2: 10.44±0.43 for -/-, osteocalcin: 113.93±10.9 for -/-, TRAP: 0.13±0.001 for -/-, cathepsin K: 0.26±0.03 for -/-, Axin2: 3.65±0.1 for -/-, β-catenin: 1.73±0.03 for -/-, LEF1: 2.49±0.38 for -/-. Statistical significance was determined by paired Student's *t*-test. * P<0.01 and ** P<0.05 versus sFRP4 +/-.

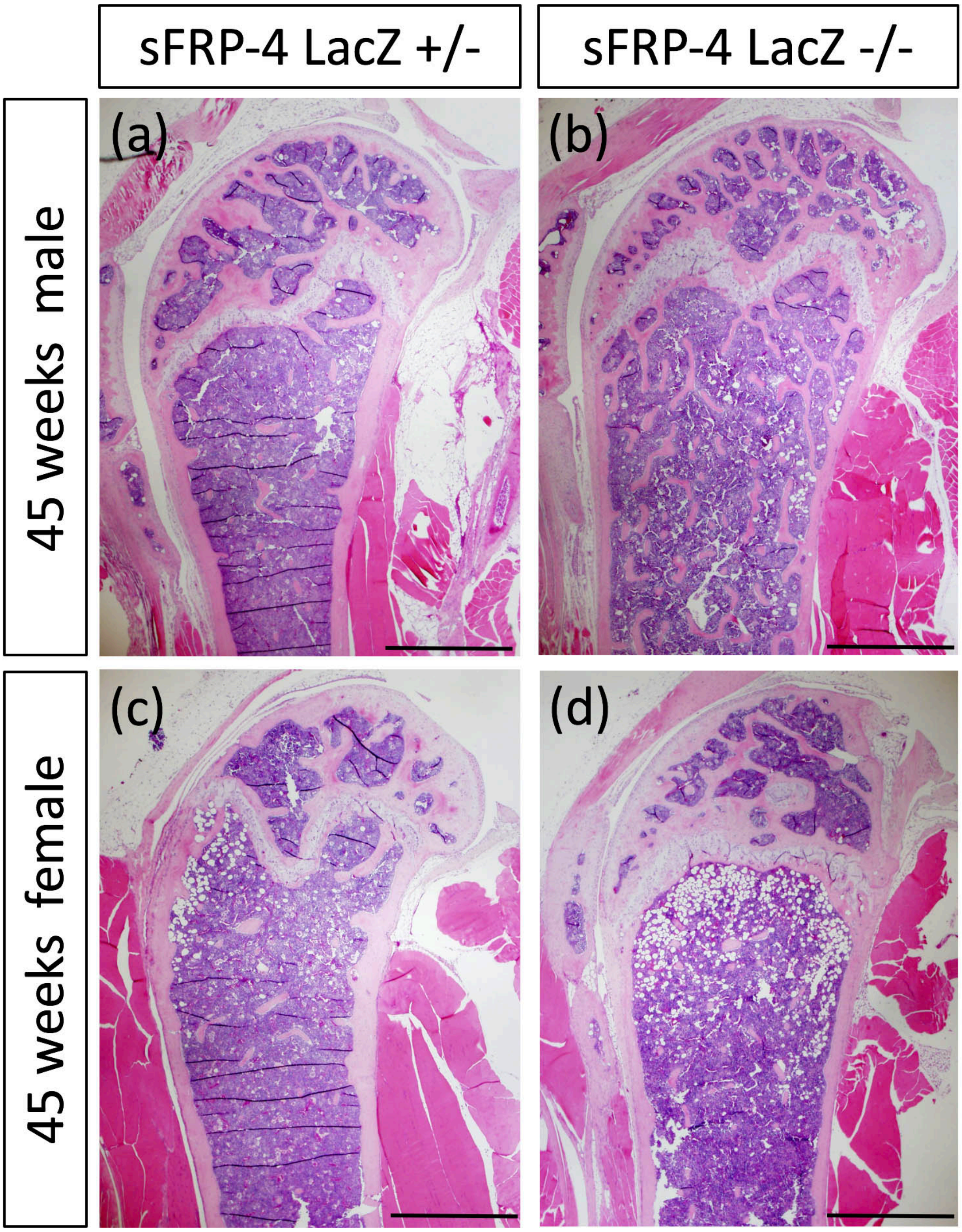

Supplementary Fig. 6. Representative micrographs of Hematoxylin and Eosin stained sections of control and sFRP4-LacZ homozygous of 45-week-old mice, related to Figure 5

The trabeculae near the proximal metaphysis in control mice are sparse and scanty compared with those in sFRP4-LacZ homozygous mice. Bars 1000μm

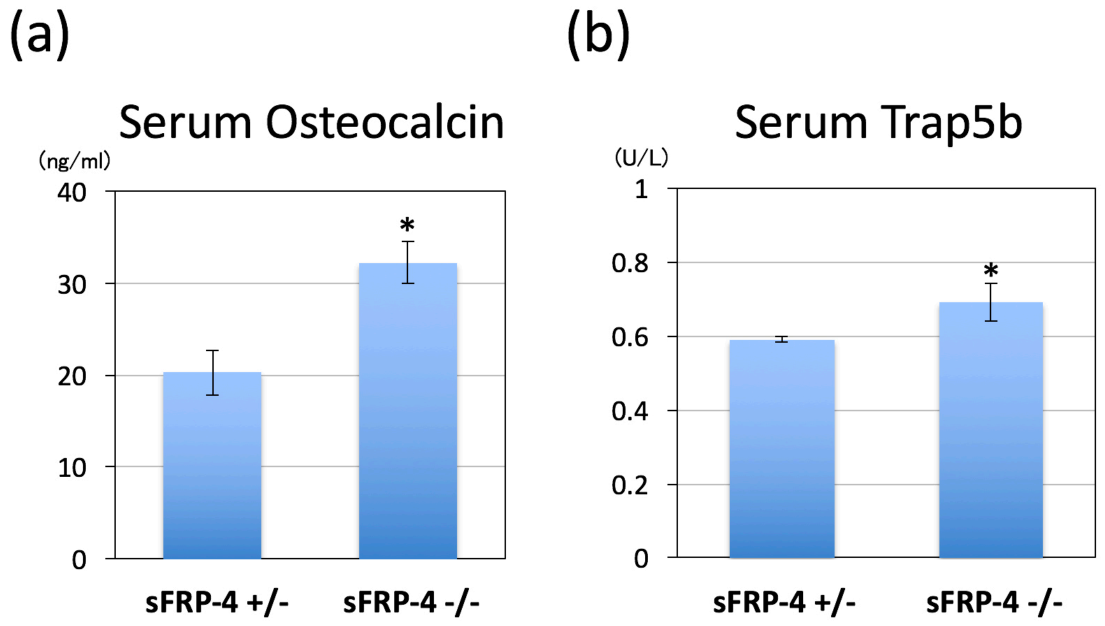

Supplementary Fig. 7. Quantification of the Serum markers of bone turnover in control and sFRP4-LacZ homozygous mice at 45 weeks, related to Figure 5

(a, b) Quantitative measurement of osteocalcin (bone formation marker) and Trap5b (as bone resorption marker) by ELISA. Error bars indicate the means ± SD (n=5); means±SD in serum osteocalcin: 20.33±2.43 for +/- and 32.28±2.27 for -/-, serum Trap5b: 0.59±0.01 for +/- and 0.69±0.05 for -/-. Statistical significance was determined by paired Student's *t*-test.* *P*<0.05 versus sFRP4 +/-.

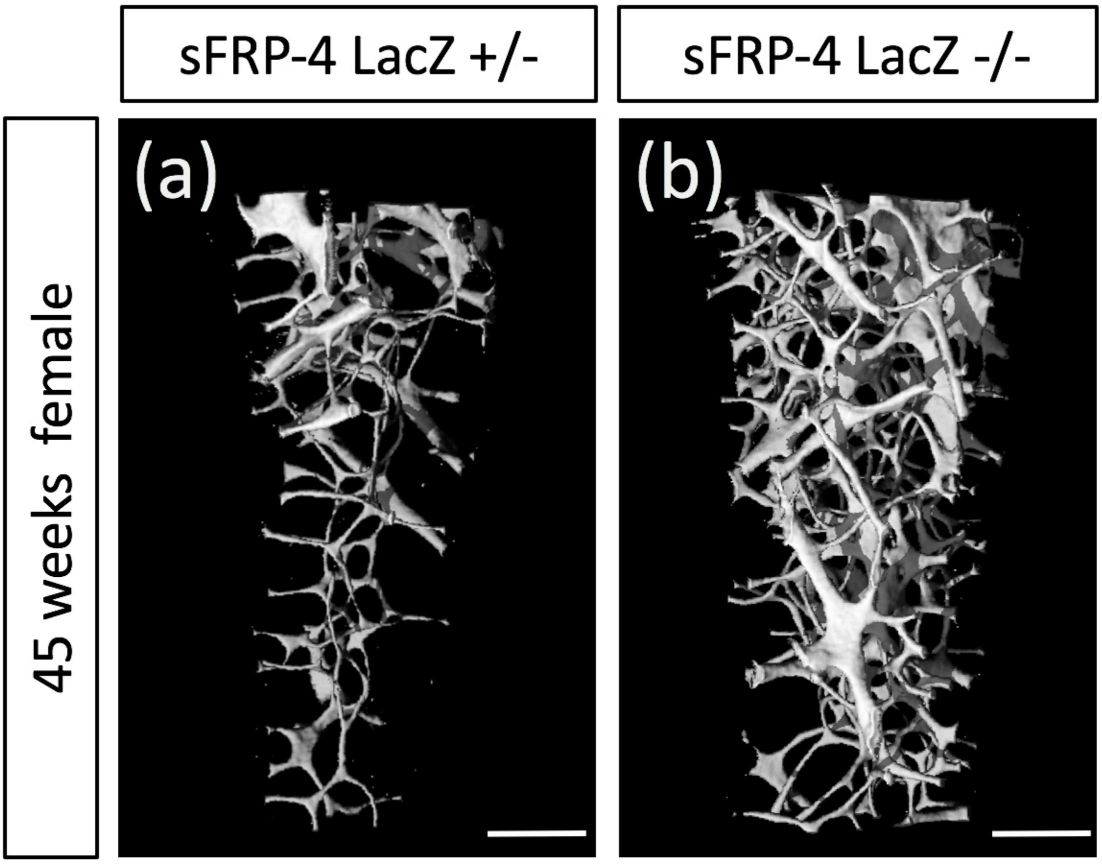

Supplementary Fig. 8. Representative μCT images of control and sFRP4-LacZ homozygous femurs of 45-week-old female mice, related to Figure 5
(a, b) Trabecular bone of the femoral region. (a) Note greatly reduced trabecular spicules indicative of trabecular osteopenia (compare with 22-week-old femur in Figure 5). (b) sFRP4 gene inactivation strikingly prevents age-related trabecular osteopenia. Bars 1200μm
